# Deciphering the action mechanism of paeoniflorin in suppressing pancreatic cancer: A network pharmacology study and experimental validation

**DOI:** 10.3389/fphar.2022.1032282

**Published:** 2022-10-20

**Authors:** Chunhao Cao, Wenting Zhao, Xianglin Chen, Bin Shen, Teng Wang, Chaoxu Wu, Xiaofeng Rong

**Affiliations:** ^1^ Department of Integrated Traditional Chinese and Western Medicine, The First Affiliate Hospital of Chongqing Medical University, Chongqing, China; ^2^ Hubei University of Chinese Medicine, Wuhan, China; ^3^ Chongqing Medical University, Chongqing, China

**Keywords:** paeoniflorin, pancreatic cancer, network pharmacology, p38 MAPK signal pathway, WGCNA

## Abstract

**Background:** Paeoniflorin (PF) is the main active component of Chinese herbaceous peony that has been shown to have an anti-tumor effect. However, there are few studies on the prevention and treatment of pancreatic cancer with PF.

**Methods:** We gathered Microarray data pertaining to paeoniflorin intervention in pancreatic cancer by utilizing the GEO database (GSE97124). Then, the DEGs were filtered by the 33R program. RNA-seq data of pancreatic cancer and normal tissue samples were taken from the TCGA and GTEx databases, respectively, and the WGCNA technique was utilized to examine the pancreatic cancer-specific genes. Paeoniflorin target genes for the treatment of pancreatic cancer were determined based on the overlap between DEGs and WGCNA. GO and KEGG enrichment analyses were then performed on paeoniflorin target genes to discover which biological processes were impacted. Using the 3 hierarchical methods included in the Cytohubba plugin, we re-screened the hub genes in the target genes to find the genes most relevant to paeoniflorin treatment. The overall survival effects of hub genes were confirmed using the TCGA database. Finally, the paeoniflorin targets identified by the network pharmacology analysis were validated using PANC-1 and Capan-2 cells.

**Results:** We identified 148 main potential PF targets, and gene enrichment analysis suggested that the aforementioned targets play a crucial role in the regulation of MAPK, PI3K-AKT, and other pathways. The further screening of the prospective targets resulted in the identification of 39 hub genes. Using the TCGA database, it was determined that around 33.33% of the hub gene’s high expression was linked with a bad prognosis. Finally, we demonstrated that PF inhibits IL-6 and IL-10 expression and p38 phosphorylation in pancreatic cancer cells, thereby reducing inflammation.

**Conclusion:** PF may regulate inflammatory factors mainly through the p38 MAPK signal pathway. These findings provide theoretical and experimental evidence suggesting the PF as a promising natural source of anti-tumor compounds for pancreatic cancer.

## 1 Introduction

Over the previous decade, the yearly number of pancreatic cancer diagnoses has increased from 43,140 to 60,430 (Jemal et al., 2010; Siegel et al., 2021). It is now the third highest cause of cancer mortality in the United States, behind lung cancer and breast cancer (Grossberg et al., 2020). Despite racial disparities in the risk of gastrointestinal disease, studies from numerous nations on various continents and with diverse ethnic structures indicate that the prevalence of pancreatic cancer continues to increase due to the aging of the global population (Jia et al., 2018; Nipp et al., 2018; Klein, 2021). Moreover, the mortality rate of pancreatic cancer is still high compared with other malignancies, and the ensuing social impact cannot be underestimated. This may be owing to the subtle start, quick development, and early metastasis of pancreatic cancer and the fact that the majority of patients have lost the window of opportunity for surgery at the time of diagnosis (Strobel et al., 2019). In addition, pancreatic cancer surgery is challenging and needs negative margins under the microscope. Only a minority of patients benefit from surgery (Kamisawa et al., 2016; Kang et al., 2016). Therefore, the great majority of patients with pancreatic cancer must receive radiation treatment or chemotherapy.

In recent years, novel targeted medicines and immunotherapy, including Erlotinib, Everolimus, and Olaparix, have offered patients some hope (Wong and Lemoine, 2009; Leroux and Konstantinidou, 2021). However, these medications and treatments continue to have many adverse events. For instance, during the administration of erlotinib, side effects such as dermatitis and diarrhea occurred with a frequency that was not negligible (Rudin et al., 2008). There have also been occasional reports of erlotinib causing interstitial pneumonia and treatment-related fatalities (Wang et al., 2016). These adverse effects have a devastating impact on older pancreatic cancer patients and may even force them to discontinue therapy. In addition, owing to individual variances, pricey targeted medications are ineffective for certain individuals. This puts the therapeutic use of some targeted medications in a dilemma. If pancreatic cancer patients are going to live longer and have a better quality of life, it is still important to find new medicines to treat the disease.

Many herbs have healing properties and have been used to treat diseases in China for thousands of years. However, it should be reminded that hazardous substances and non-pharmaceutical chemicals are also present throughout the herb. Therefore, it is a reasonable choice to investigate natural substances with well-defined chemical structures that are isolated from herbal remedies as medicinal pharmaceuticals. Numerous natural compounds, like paclitaxel, resveratrol, etc., have shown potent anticancer activity. Even at large concentrations, several natural chemicals are well tolerated by patients (Rejhová et al., 2018). Therefore, novel natural chemicals have a promising future in the creation of anti-cancer medications. Paeoniflorin (PF) is the main active component of Radix Paeoniae Alba, Radix Paeoniae Rubra, and Paeonia Suffruticosa Andr, which is a water-soluble monoterpene glycoside (Wu et al., 2010), extracted from the peony in 1963 for the first time (Hu et al., 2013). By blocking the activity of the Notch-1 signaling pathway, Zhang and colleagues have shown that PF reduces the growth and invasion of breast cancer cells (Zhang et al., 2016b). Treatment of colorectal cancer cells with PF leads to downregulation of FoxM1 and inhibits colorectal cancer cell migration (Yue et al., 2018). However, the chemical and pharmacological foundation of PF as a pancreatic cancer inhibitor has not been proven or investigated.

**FIGURE 1:**
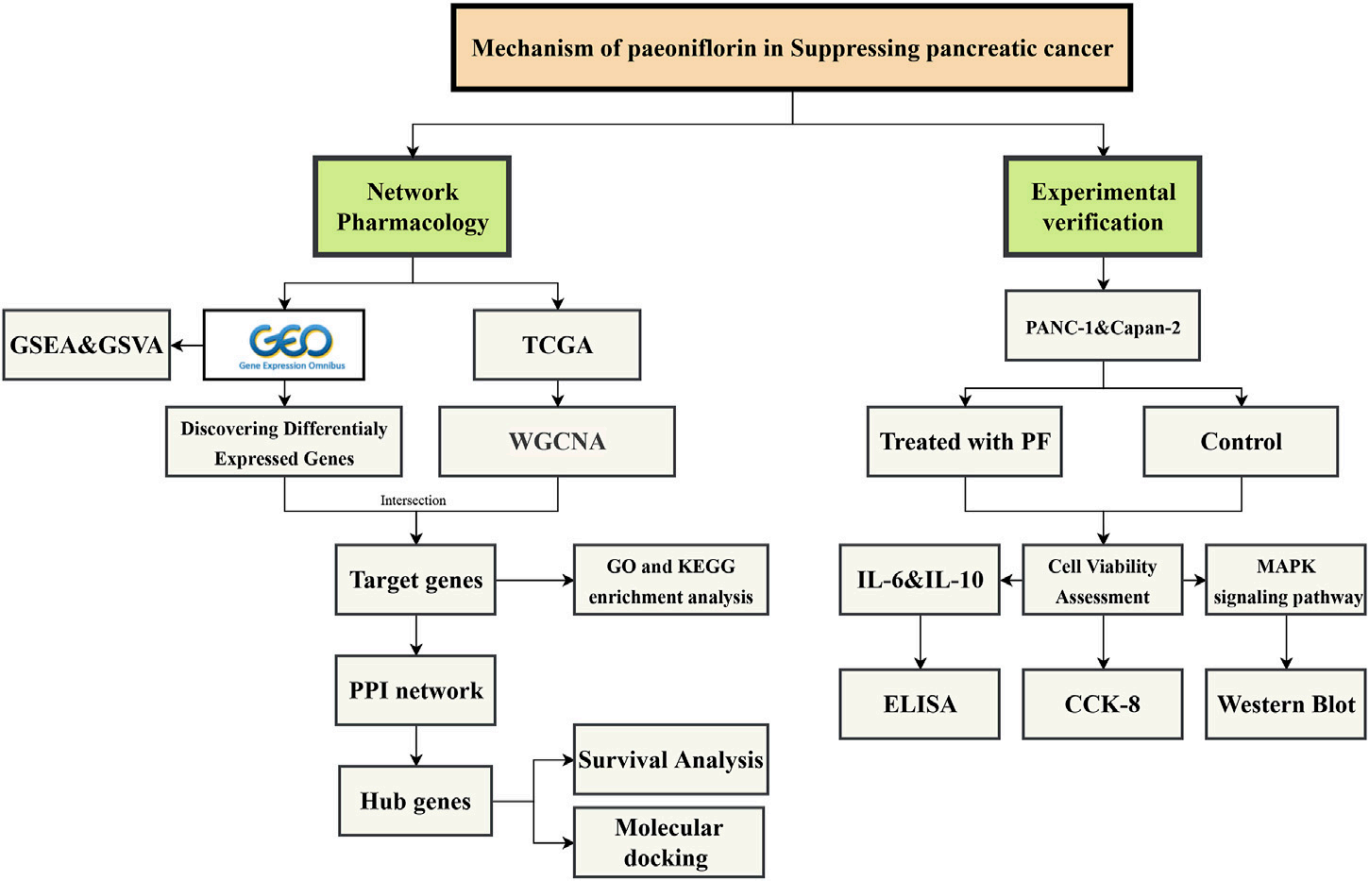
The workflow of this study.

Network pharmacology is a novel inter-disciplinary technique that has assisted several researchers in investigating the pharmacological effects of natural substances and compound herbal remedies. To further elucidate the mechanism of action of PF in the treatment of pancreatic cancer, we used network pharmacology to investigate the impact of PF on pancreatic cancer and confirmed the regulatory link between PF and key signaling pathways *in vitro*. Figure 1 depicts the workflow for this research.

## 2 Materials and methods

### 2.1 Predicting the targets genes of the paeoniflorin in pancreatic cancer

GSE97124 (Li et al., 2017) is a dataset related to paeoniflorin treatment of pancreatic cancer from the GEO database (http://ww.ncbinlm.nih.gov/geo/). The dataset for pancreatic cancer was preprocessed and normalized for future analysis using the normalize Quantities function of the limma (Ritchie et al., 2015) package in R (Version 4.1.0). Using the limma, differentially expressed genes (DEGs) were assessed between paeoniflorin-free and paeoniflorin-treated samples. The results are plotted with the ggplot2 package as a volcano map and heatmap (Ito and Murphy, 2013).

Weighted gene correlation networks Analysis (WGCNA) reveals modules of co-expressed genes in complex biological procedures (Langfelder and Horvath, 2008). For WGCNA analysis, we utilized 177 pancreatic cancer tissues from The Cancer Genome Atlas (TCGA, https://portal.gdc.cancer.gov/) and 167 normal tissues from Genotype-Tissue Expression (GTEx, https://commonfund.nih.gov/GTEx). Confirm the soft threshold using the pickSoftThreshold function of the WGCNA package in R with reference to Linbang et al. (Wang et al., 2021). Following the formation of a scale-free network according to the soft threshold, a topology matrix and hierarchical clustering are applied. That meant 60 genes were the bare minimum per module needed for dynamic gene module cleavage. Then, each module’s Eigengenes were established. Based on the Eigengenes module, hierarchical clustering was carried out once the correlation between modules was established. A total of six modules were created by once more combining the earlier components. The link between modules and between modules and PF was investigated using Pearson correlation. Significantly connected modules were regarded as crucial PF elements for a subsequent investigation.

Finally, paeoniflorin’s target genes for the therapy of pancreatic cancer were determined by looking at the overlap between WGCNA and DEGs screening.

### 2.2 Gene set enrichment analysis and Gene Set Variation Analysis

Many of the most enriched gene sets defining metabolic activities were uncovered using GSEA (Subramanian et al., 2005). We ran a gene set enrichment analysis on Microarray data collected from the GEO database to investigate the changes induced by paeoniflorin. The gene set “c2. cp.v7.2. symbols.gmt” from the MSigDB database (https://www.gsea-msigdb.org/gsea/msigdb/) was used for the aforementioned procedures (Liberzon et al., 2015). The R package “GSVA” was used to perform Gene Set Variation Analysis (GSVA). An enrichment score (ES) was generated for each sample and pathway as a result of the analysis using a non-parametric unsupervised approach that converted a traditional gene matrix (gene-by-sample) into a gene set-by-sample matrix. Then, the mean values of ES of cells in the two groups of samples were compared using the *t*-test. The MSigDB database’s “c2. cp.kegg.v7.5.1. symbols” is one of the target gene sets used in this study. A false discovery rate (FDR) < 0.25 was considered significant enrichment (Zhang et al., 2016a).

### 2.3 Gene ontology and kyoto encyclopedia of genes and genomes enrichment analysis

Gene ontology (GO) enrichment analysis and Kyoto Encyclopedia of Genes and Genomes (KEGG) enrichment analysis were performed on target genes (Gene Ontology Consortium, 2015; Kanehisa et al., 2019). The “clusterProfiler” package (Yu et al., 2012) of the R program was used to carry out these gene enrichment analyses. Pathways with *p* < 0.01 were considered statistically significant. Then, the visualized the top 20 or 30 pathways using the “ggplot2” package in R.

### 2.4 Building a PPI network and Kaplan-Meier curves

The Protein-Protein Interaction Network was built by inserting target genes into the STRING database (Szklarczyk et al., 2017). Change the needed minimum interaction score to 0.9. The PPI network was visualized and displayed by Cytoscape (Shannon et al., 2003). Likewise, the cytoHubba plugin (Shannon et al., 2003) was used to exclude non-central genes from the target genes, substantially reducing the number of target genes. We then used the TCGA database to examine the association between the expression of these hub genes and prognosis. The specific strategy involves downloading the pancreatic cancer dataset from the TCGA database through the TCGAbiolinks package (Colaprico et al., 2016), and then choosing a total of 178 individuals with complete overall survival (OS) and clinical characteristics. Patients were divided into high- and low-expression groups based on the median of the difference in expression of a single hub gene; the difference in overall survival between the two groups was then examined using the Kaplan-Meier curves. The “survminer” package is utilized for data visualization, whereas the “survival” package is utilized for survival data statistical analysis.

### 2.5 Molecular docking verification

Molecular docking was utilized to anticipate interactions between paeoniflorin and its primary targets. The Protein Data Bank (Berman et al., 2000) contains detailed information and 3D structures, including the original structures of important targets. Paeoniflorin’s chemical structure was derived from the PubChem website (Kim et al., 2021). Import the structure of the aforementioned protein into the AutoDock Vina tool (Seeliger and de Groot, 2010), select the default settings, and set the Grid Box to the entire protein molecule. Then, run AutoDock Vina for molecular docking to confirm the binding activity of the target and the compound and obtain the binding energy.

### 2.6 Experimental validation *in Vitro*

#### 2.6.1 Reagents and materials

Prior to usage, the purity of paeoniflorin (CAS: 23180-57-6) acquired from Sigma-Aldrich (St. Louis, United States) was verified by UPLC-MS to be more than 95%. From Procell Co., Ltd. (Wuhan, China) purchased the human pancreatic cancer cell lines PANC-1 and Capan-2. Gibco supplied the DEME, fetal bovine serum, and penicillin-streptomycin necessary for cell culture (Grand Island, United States). From BOSTER Technology Co., Ltd. (Wuhan, China) bought ELISA kits. DMSO, CCK-8 kit, RIPA buffer, and BCA Protein Assay Kit were procured from Solarbio Technology Co., Ltd. (Beijing, China). Primary antibodies against p38, phospho-p38, p44/42 MAPK (Erk1/2), phospho-p44/42 MAPK (Erk1/2), SAPK/JNK, phospho-SAK/JNK, and β-actin were bought from Abcam and Cell Signaling Technology, both in the United States.

#### 2.6.2 Cell culture and viability assay

Panc-1 cells and Capan-2 were cultured in a 10% FBS and 100 U/mol penicillin-streptomycin solution in an incubator at 37°C, 5% CO_2_, and 95% relative humidity. Upon intervention, every cell was in the logarithmic growth phase. PF was dissolved in DMSO to achieve a concentration of 400 μM. 100 ml of PF (400 μM) was then diluted to 200, 100, and 50 μM, respectively, in 100, 300, and 700 ml of DMEM. In six-well plates, cells (about 1 × 10^6^ per well) were seeded and cultured for 24 h with varying doses of PF-treated DEME or DEME without PF. The collection of cells and cell culture media for use in later investigations. CCK-8 cell viability was evaluated in accordance with the manufacturer’s instructions. Panc-1 cells were planted at a density of 3 × 10^4^ cells per well in 96-well plates (approximately 100 μl medium per well). After 24 h of treatment with various PF concentrations, 10 μl of CCK-8 solution was applied to the 96-well plate. The plate was incubated at 37°C for 4 h. Use a microplate reader (Bio-Rad, United States) to figure out the optical density of each well at 450 nm.

#### 2.6.3 Measurement of IL-6 and IL-10

Using an ELISA kit and the manufacturer’s instructions, the IL-6 and IL-10 levels in cell culture supernatants were assessed.

#### 2.6.4 Western blot analysis

After 48 h of treatment with various doses of PF (0, 50, 100, and 200 μM), PANC-1 cells were harvested. PANC-1 cells were lysed in RIPA buffer with a protease and phosphatase inhibitor cocktail. Upon completion of the lysis, the protein concentration was determined with the BCA kit. 30 mg of protein was loaded per lane on 10% SDS-PAGE gels and transferred to 0.45 m PVDF membranes (Millipore). After 60 min of blocking with 5% skim milk, the membrane was incubated at 4°C overnight with the primary antibody (1:1000) indicated in 2.6.1. After three washes, the membrane was re-incubated with HRP-conjugated secondary antibody IgG (1:2000) at room temperature for 1 h in the dark. Signals were recognized by autoradiography after applying ECL (FluorChem E, Proteinsimple, United States). The densitometric findings were examined using ImageJ (National Institutes of Health, United States).

### 2.7 Statistical analysis

The information was given in the form of means and standard deviations. A student’s t-test was used to assess differences between pairs of groups, while one-way analysis of variance (ANOVA) was used to investigate differences between three or more groups. Data analysis and graphing were performed using GraphPad Prism 7 (San Diego, United States).

## 3 Results

### 3.1 Identification of Target genes in datasets

**FIGURE 2:**
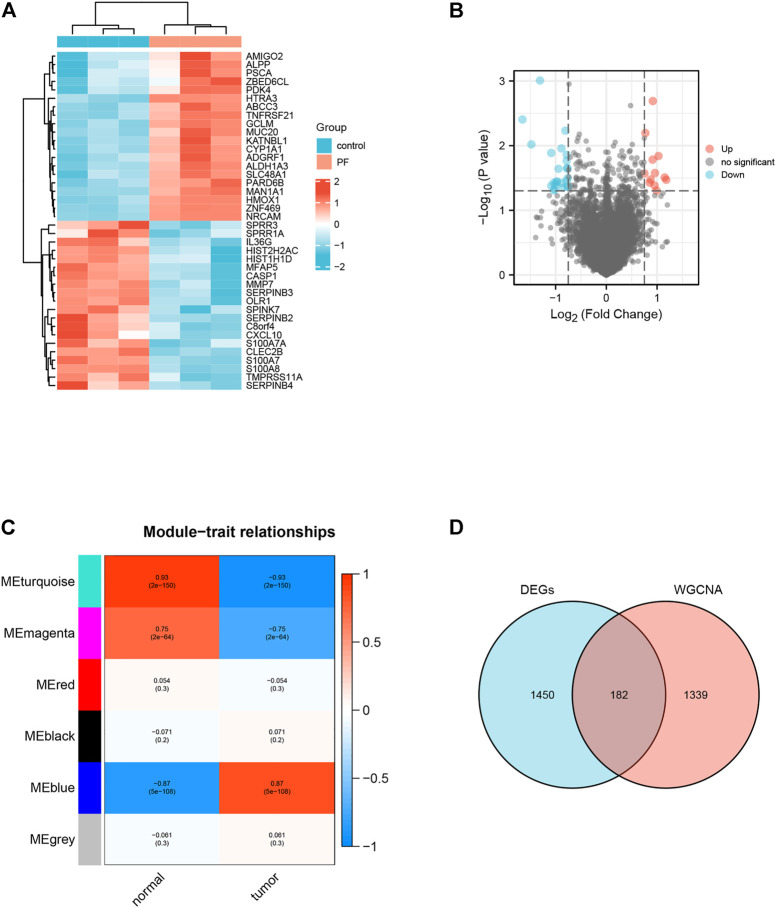
Identification of Target genes for paeoniflorin treatment of pancreatic cancer. **(A)** Heatmap of the top 40 DEGs associated with Paeoniflorin-treated pancreatic cancer. **(B)** The Volcano plot shows the distribution of genes that are differentially expressed (log2 fold change) compared to a measure of statistical significance (−log10 *p*-value) in the GSE97124 dataset. Genes that were downregulated are shown in blue, whereas genes that were upregulated are shown in red. **(C)** The results show a highly significant correlation between GS and MM in the MEblue module. **(D)** Genes screened by DEGs (pink circles), genes in modules strongly connected to PF in WGCNA analysis (blue circles), and portions of these two classes of genes that overlap (purple circles).

We identified 1632 differentially expressed genes (DEGs) in the GSE97124 dataset based on a *p* value <0.01 (Figures 2A,B). Next, we did an analysis of the TCGA and GTEx data using the WGCNA method. The pickSoftThreshold function identifies 9 as the optimal soft threshold (Supplementary Figure S1A). Then, the dynamic tree cutting algorithm identifies six modules, which are differentiated by distinct colors and marked on the diagram (Supplementary Figures S1B,C). Module eigengenes (ME) refer to the major components of every module. By evaluating the correlation between the modules’ eigengenes, it was evident that the MEblue modules were most strongly related to pancreatic cancer (Supplementary Figure S1D; Figure 2C). Therefore, we considered the genes in the MEblue module as disease-related characteristic gene sets. The MEblue module has 1521 genes, of which 182 (11.97%) overlap with DEGs (Figure 2D). Select as target genes the genes that overlap between DEGs and this module gene.

### 3.2 GSEA and GSVA

**FIGURE 3:**
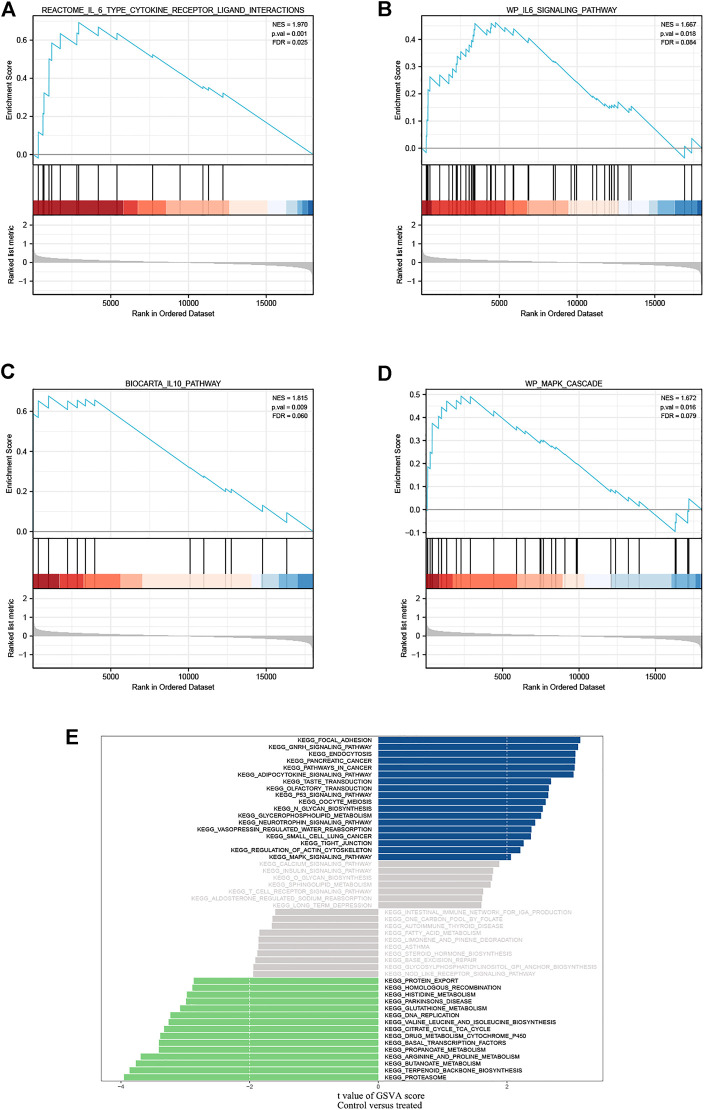
GSEA identified IL-6 type cytokine receptor ligand interactions. **(A,B)** IL-6 family signaling pathways; **(C)** IL-10 pathway and **(D)** MAPK cascade as regulatory targets of paeoniflorin in pancreatic cancer. **(E)** Differences in pathway activities scored per sample by GSVA between PF-treated group and control group. Blue represents the pathway with t value greater than 2, and green represents the pathway with t value less than -2. NES, normalized enrichment score; FDR, False discovery rate.

Using the previously mentioned R software package, we investigated significantly enriched pathways for paeoniflorin intervention and control groups. In the Microarray data set, IL-6, IL-10, and MAPK pathways were strongly related to paeoniflorin intervention, indicating that paeoniflorin may play a crucial role *via* these pathways (Figures 3A–D). We also performed a GSVA analysis. The results of a GSVA analysis were arranged by the magnitude of the absolute t-value and displayed in figure format (Figure 3E). In terms of biological processes such as focal adhesion, gnrh signaling pathway, endocytosis, proteasome, terpenoid backbone biosynthesis, and butanoate metabolism, there were substantial variations between the treatment group and the control group.

### 3.3 Biological Function and Pathway Analysis

**FIGURE 4:**
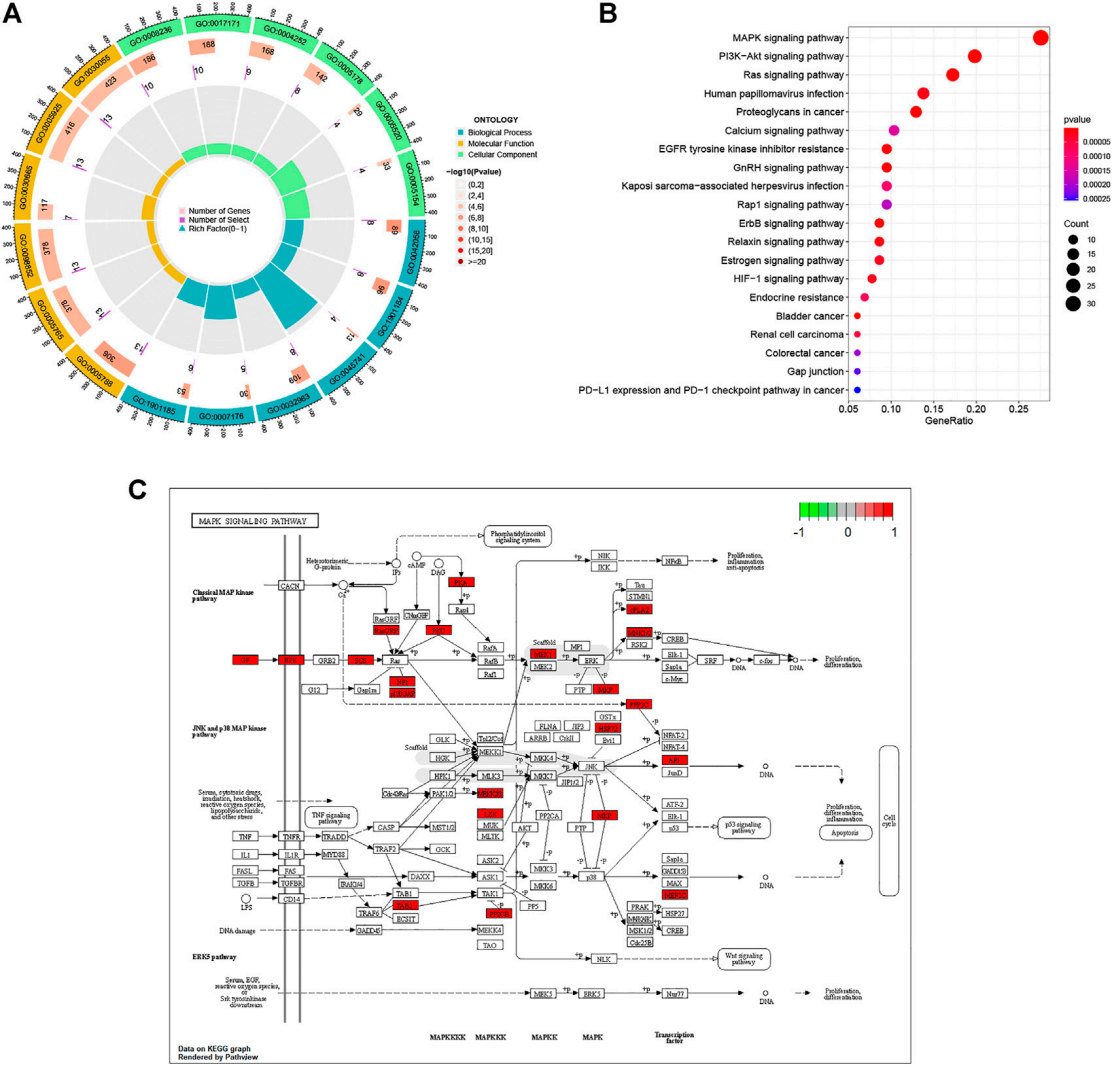
Biological Function and Pathway Analysis. **(A)** GO enrichment analysis of target genes. In the figure, terms enriched in the BP category are depicted in blue, terms enriched in the CC category in green, and terms enriched in the MF category in yellow. The *p* value’s cutoff value was set at 0.05. *p* value is used to rank terms within a category. **(B)** KEGG enrichment analysis of target gene. **(C)** Distribution of Target genes in the MAPK signaling pathway. The red box in the figure represents Target genes.

We utilized the “clusterProfiler” package to do GO annotation and KEGG pathway analysis on pancreatic cancer target genes. Figure 4A; Supplementary Table S1 depict the most enriched items across the three categories of biological process (BP), cellular component (CC), and molecular function (MF). Epidermal growth-related pathway, ERBB signaling pathway, collagen metabolic process, ribosome, and regulation of protein tyrosine kinase activity were among the targets in the BP test. The four most frequently occurring GO terms under the category of cellular component were endoplasmic reticulum lumen, lysosomal membrane, lytic vacuole membrane, and clathrin-coated vesicle membrane. The overwhelming majority of CC terms were linked to vesicles or membranes. In MF, the primary targets were serine-type peptidase activity, serine hydrolase activity, serine-type endopeptidase activity, and integrin binding. Based on a *p*-value of 0.05, KEGG analysis identified 50 enriched pathways (Figure 4B, Only the top ten enriched terms are shown in the figure). This suggests that paeoniflorin’s regulatory mechanisms include the PI3K-AKT signaling route, the RAS signaling pathway, and the MAPK signaling network. Since the MAPK signaling route was included in both GSEA, GSVA and KEGG enrichment, we visualized the link between important genes and the MAPK signaling pathway (Figure 4C).

### 3.4 PPI and survival analysis

**FIGURE 5:**
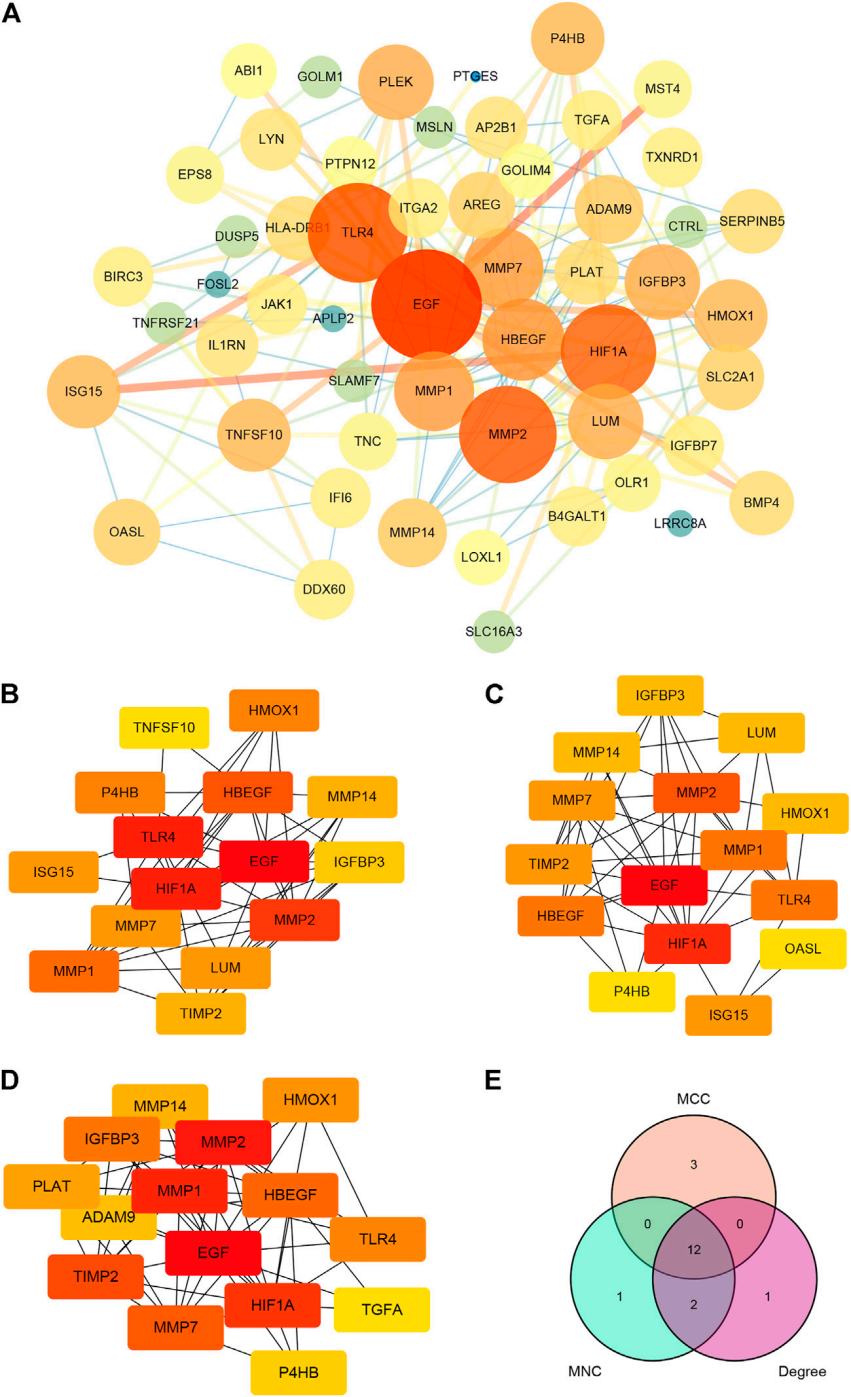
PPI network construction and hub genes screening. **(A)** PPI network of paeoniflorin for treating pancreatic cancer. The greater the number of linked nodes, the larger and darker the circle and its color. Three screening techniques for hub genes (implemented by the Cytohubba plugin). Yellow nodes have the lowest correlation strength, whereas red nodes have the greatest. **(B)** MCC; **(C)** MNC; **(D)** DEGREE. **(E)** A Venn diagram analysis of hub gene screening using Cytohubba. Each hue corresponds to a screening algorithm. The core gene is the hub gene, which is the gene present in all five algorithms.

Import the 184 targets discovered in 3.1 into the STRING platform, set the confidence score to 0.9, and construct a PPI network. Due to the enormous number of nodes, the 182 target genes with the highest node values are displayed on the graph There are 126 nodes in the PPI network and 238 edges (as shown in Figure 5A, the color gradually turns yellow, the greater the possibility of becoming a core protein). The Cytohubba plugin includes the following degree algorithms: MNC, MCC, and DEGREE. We implement these algorithms to filter nodes in the PPI network and designate the intersection nodes as hub genes (Figures 5B–D). The conclusion is represented by a Venn diagram (Figure 5E). These hub genes are predominantly associated with the matrix metalloprotein (MMP) family. The above findings show that paeoniflorin may have a role in the therapy of pancreatic cancer by interfering with these main targets.

**FIGURE 6:**
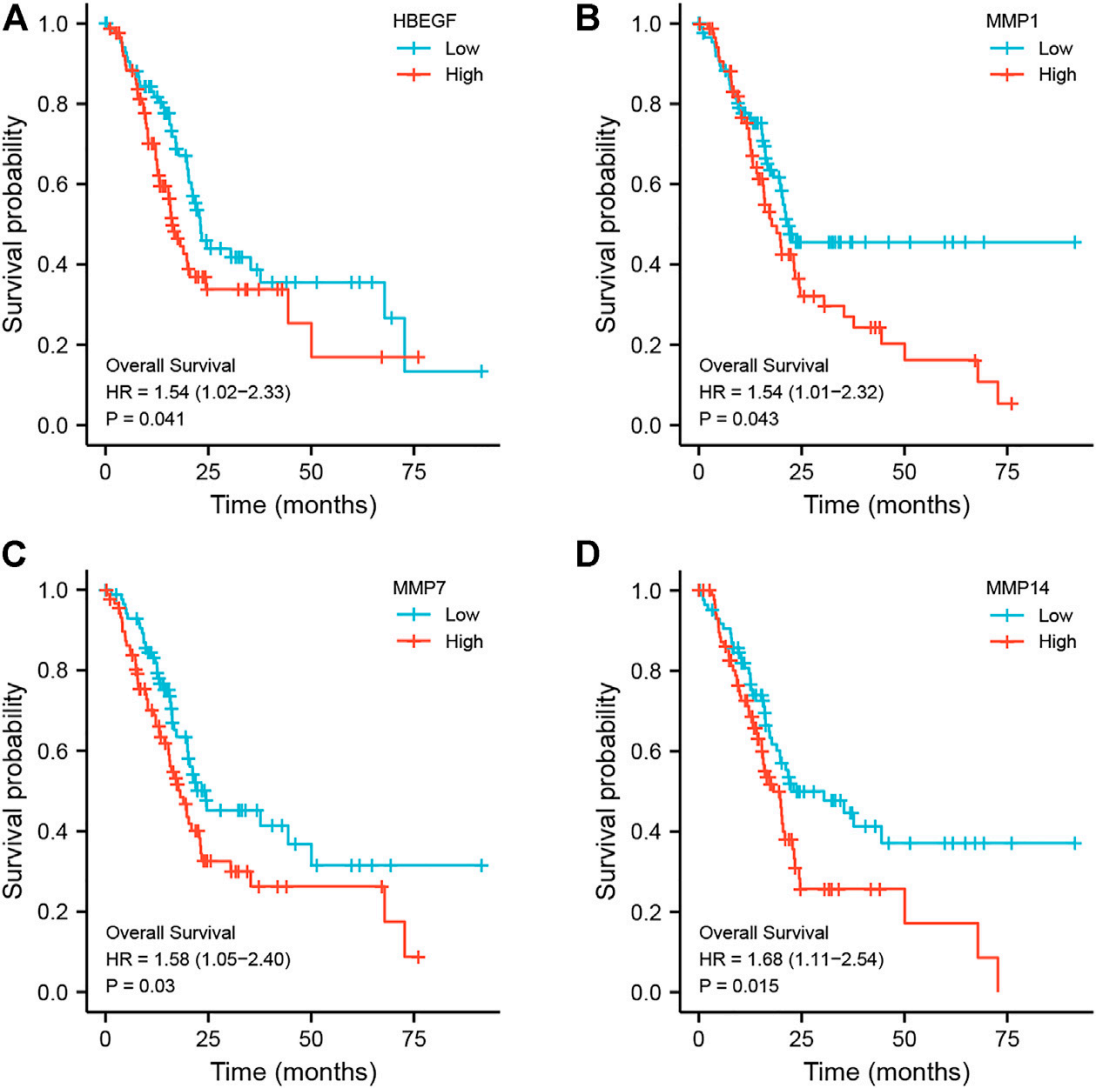
Kaplan-Meier curves of hub genes. According to the expression level of each hub gene, it is separated into high expression (red line) and low expression (blue line) groups, and the KM curve of each gene is drawn independently. **(A)** HBEGF; **(B)** MMP1; **(C)** MMP7; **(D)** MMP14.

**FIGURE 7:**
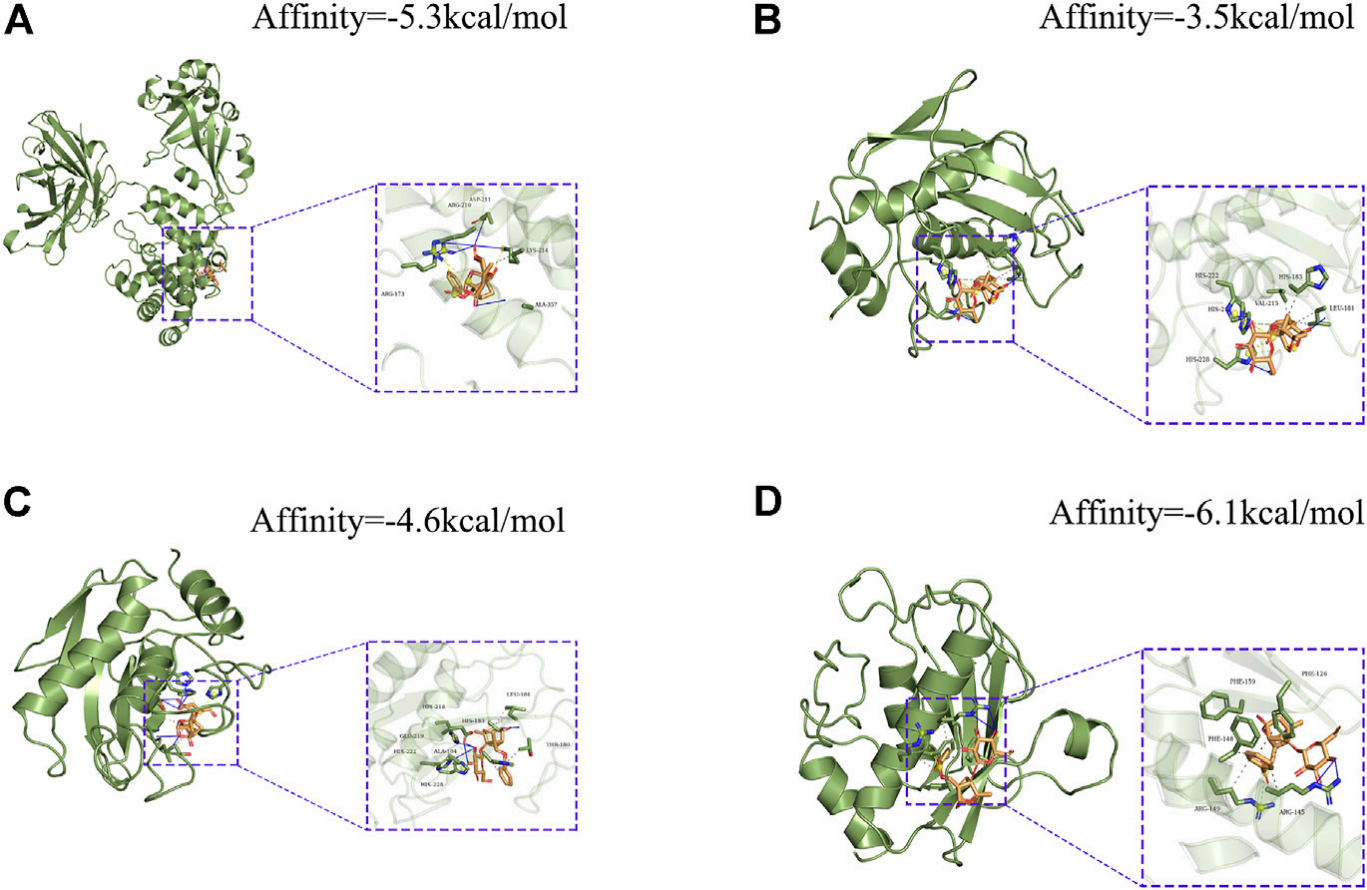
Molecular docking models of PF binding to potential targets. **(A) **HBEGF; **(B) **MMP1; **(C) **MMP7; **(D) **MMP14.

In addition, using pancreatic cancer patient information from the TCGA database, we analyzed the effect of these hub genes on overall survival. Four of the twelve genes had clearly separated KM curves. This indicates that low expression of these four genes (MMP1, MMP7, MMP14, and HBEGF) predicts longer overall survival than high expression (Figure 6). These four genes and PF were subsequently considered ligands and receptors, respectively. Lower binding energies suggest a more stable binding configuration between the receptor and ligand. Figure 7 depicts interactions between receptors and their ligands and affinity. The image on the left was captured when the lens was focused further away, while the one on the right was captured when the lens was focused closer.

### 3.5 PF inhibited proliferation of pancreatic cancer cells

**FIGURE 8:**
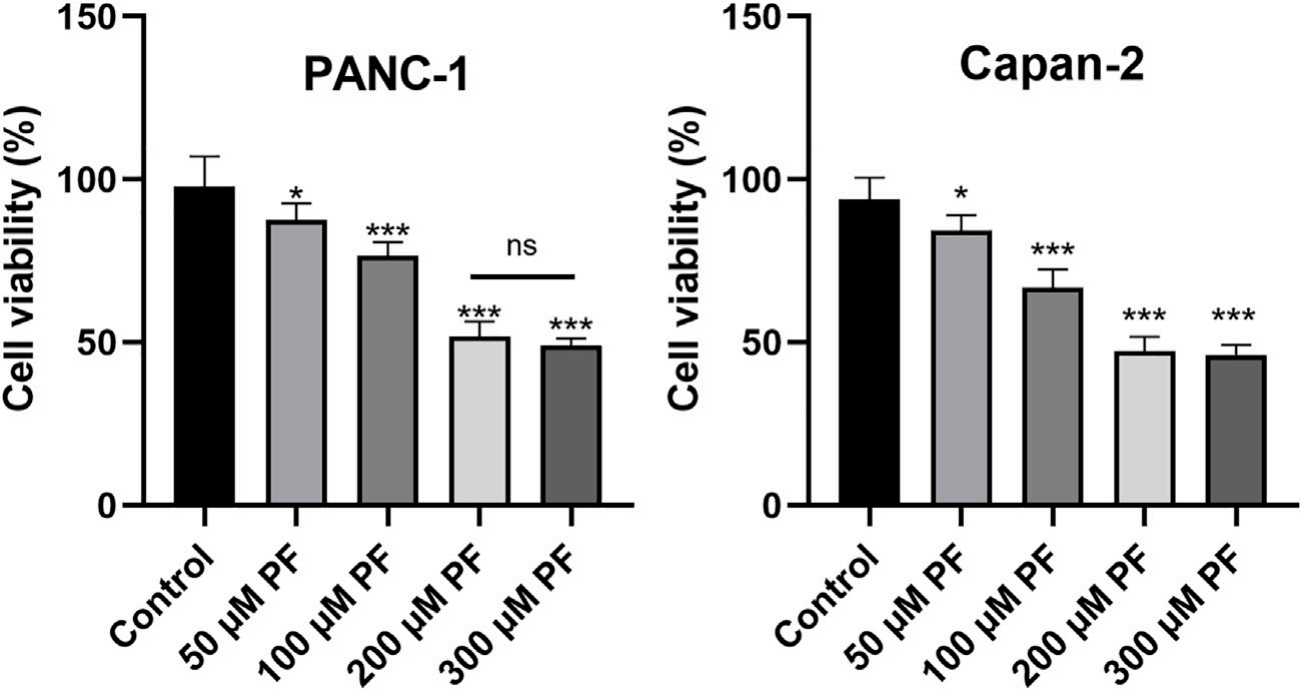
Effects of PF intervention on the growth of pancreatic cancer cells. PANC-1 cells and Capan-2 were treated with 50 μM, 100 μM, 200 μM, and 300 μMPF, and the CCK-8 assay was used to evaluate cell viability. The experiments were repeated at least three times. Data were expressed as mean ± SD. *p < 0.05; ***p < 0.001, *versus* control group.

PANC-1 and Capan-2 cells were treated with PF (0, 50, 100, 200, and 300 μM) for 24 h to determine the effects of various PF concentrations on human pancreatic cancer PANC-1 and Capan-2 cells. The findings demonstrated that the presence of PF lowered the number of viable PANC-1 and Capan-2 cells in comparison to the control group (Figure 8). In addition, PF inhibited both types of pancreatic cancer cells in a concentration-dependent manner between 0 and 200 μM. The inhibitory effect did not change appreciably when the concentration of PF was increased to 300 μM from 200 μM. In the subsequent trials, dosages ranging from 0 to 200 μM are used.

### 3.6 PF alleviates inflammation in PANC-1 cells and Capan-2 cells

**FIGURE 9:**
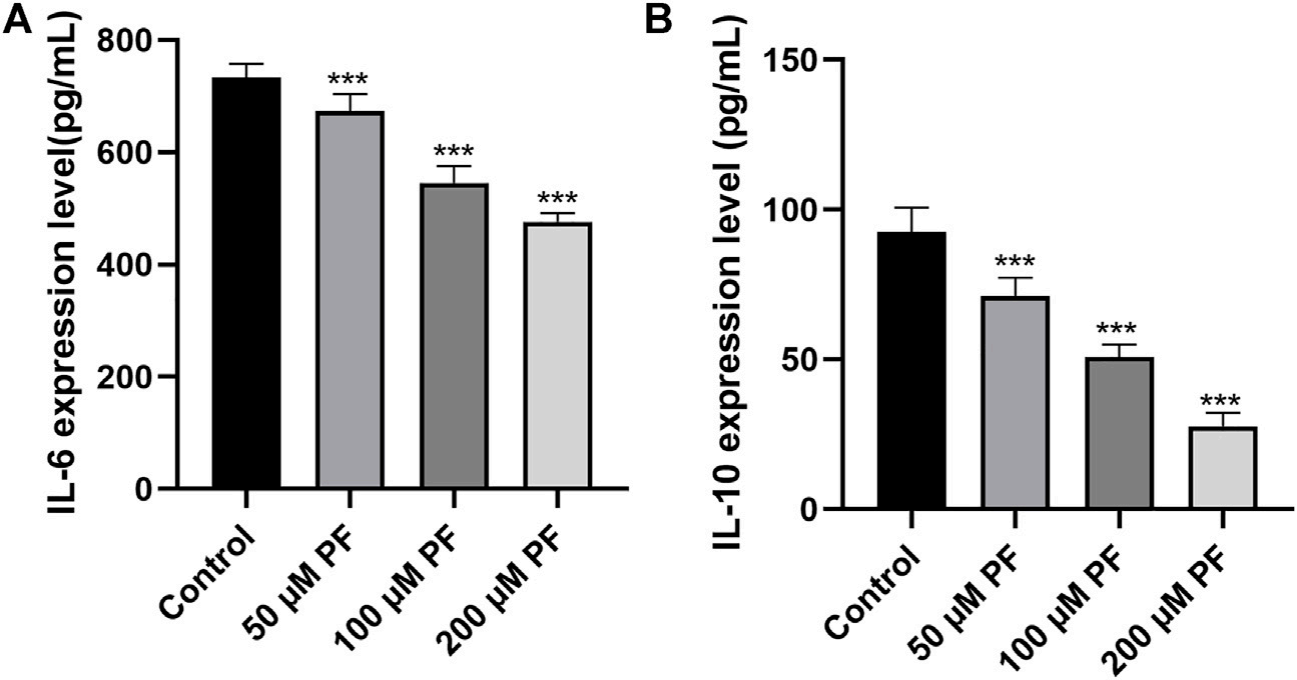
The contents of IL-6 and IL-10 in the culture supernatant of pancreatic cancer cells were determined by ELISA. **(A)** IL-6 expression in the culture supernatant of PANC-1 cells. **(B)** IL-10 expression in the culture supernatant of Capan-1 cells. *p < 0.05; ***p < 0.001, *versus* control group.

The levels of inflammatory factors were measured to establish whether PF may attenuate the inflammatory response in pancreatic cancer. ELISA showed that the levels of IL-6 and IL-10 were lower in the group that was given PF than in the control group (Figure 9).

### 3.7 PF suppressed the MAPK signal pathway

**FIGURE 10:**
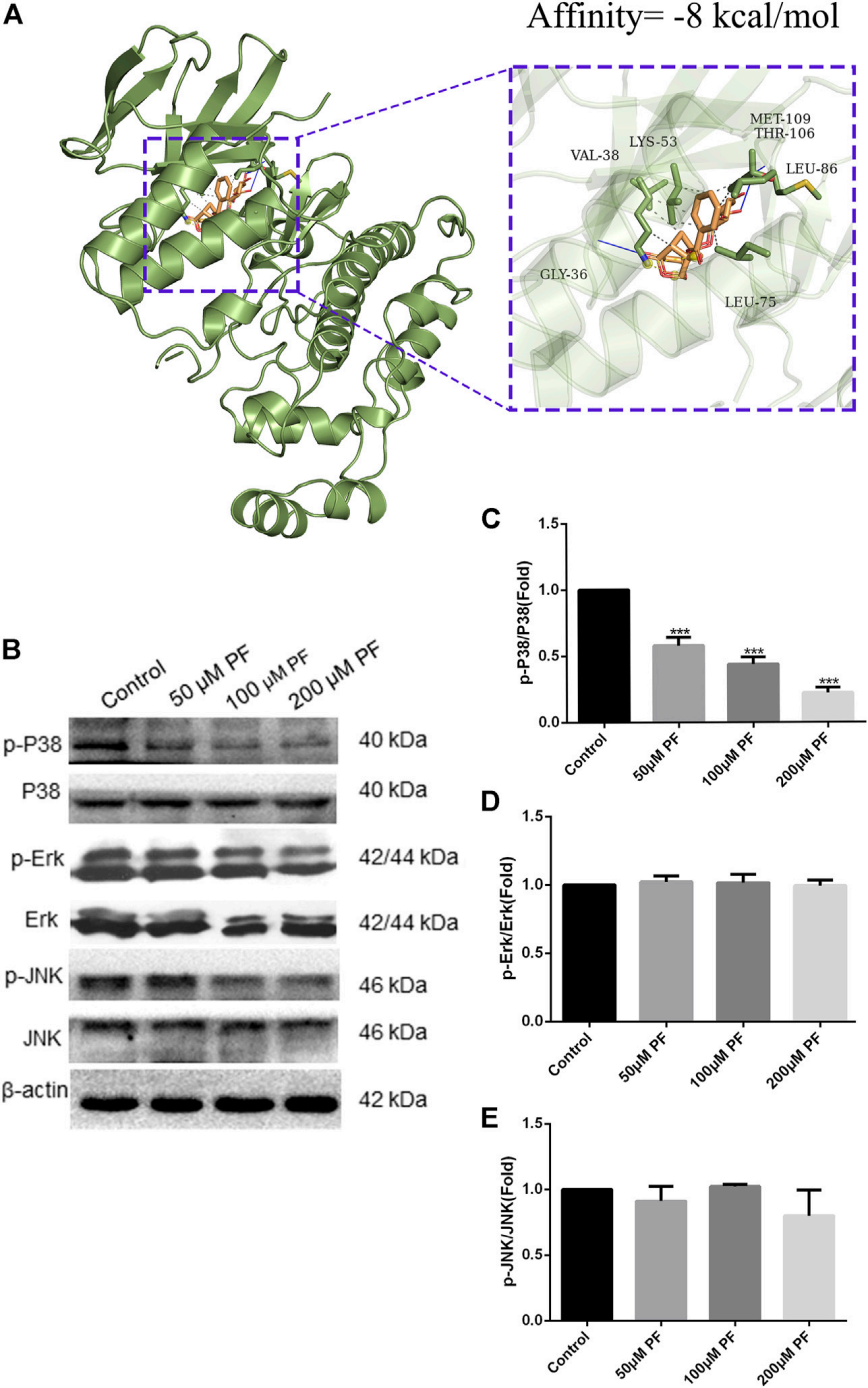
PF suppressed MAPK signal pathway in PANC-1. **(A)** PF exhibited good binding activity to p38 as determined by molecular docking. **(B)** Based on the results of Western blot analyses, PF was shown to decrease p38-related phosphorylation in MAPK signaling. This image displays some typical consequences from WB banding. **(C–E)** Each protein’s relative expression level was measured using statistical methods: p-ERK, p-JNK, and p-P38. ***p < 0.001 vs. control group.

Firstly, we conducted docking experiments between PF and p38. Figure 10A depicts the final output optimization using AutoDock Vina. Docking data indicated that the absolute value of the affinity of the target protein for PF is 8. The lower the binding energy, the greater the binding activity and the greater the target protein’s capacity to bind to PF. Consequently, p38 can tightly bind to PF. Subsequently, we demonstrated the therapy of pancreatic cancer with PF through the MAPK pathway in pancreatic cancer cells. In the range of 0–200 μm, the inhibitory impact of PF on p38 phosphorylation grew progressively, but p38 concentrations did not differ substantially from those of the controls. PF reduced the activation of P38 MAPK, but not ERK and JNK phosphorylation, as revealed by a standard western blot (Figures 10B–E).

## 4 Discussion

Due to the high lethality of pancreatic cancer, it is vital to create new therapies and medications for pancreatic cancer. It should be highlighted that the molecular process underlying the formation and progression of pancreatic cancer is complicated, including numerous proteins or pathways, and therefore a single targeted treatment may not have the desired therapeutic impact (Li et al., 2019). According to previous studies, a number of Chinese medicines and herbal extracts offer unique advantages in the treatment of pancreatic cancer (Triantafillidis et al., 2022). Quercetin, for instance, may block EMT by reducing TGF-β, cause cell death, and reduce the development of pancreatic cancer cells by downregulating c-Myc expression (Asgharian et al., 2021). Apigenin contains antioxidant and anti-inflammatory characteristics and can exert a therapeutic effect on PC cells *via* HIF, VEGF, and GLUT-1 (Ashrafizadeh et al., 2020). The medicinal effects of the chemicals derived from these plants are multitarget and multipathway. Then we set our sights on paeoniflorin, a natural compound with anti-inflammatory and anti-tumor effects (Zhou et al., 2020; Wang et al., 2022). Paeoniflorin has been demonstrated to inhibit the early phases of EMT induced by TGF-β. This may be accomplished by inhibiting the production of transcription factors Snail and Slug *via* the Smad pathway (Ji et al., 2016). Paeoniflorin impacts the progression of hepatocellular carcinoma, which is also a cancer of the digestive system, by downregulating the 5-HT1D inhibitory Wnt/β-Catenin pathway (Zhou et al., 2021). Moreover, pancreatic cancer growth is inhibited by paeoniflorin, which does so through increasing HTRA3 (Li et al., 2017). So, we used network pharmacology to look into the possible pharmacological mechanism of paeoniflorin, thinking of it as a possible molecule for treating pancreatic cancer.

Previous network pharmacology is defined by the use of several internet databases to construct a network of multiple links between medications, targets, and disorders in order to study drug pharmacology (Jiao et al., 2021). For paeoniflorin, a substance isolated from plants with a well-defined chemical structure and characteristics, the usage of pharmacological data retrieval targets cannot be used to create an intersecting network. In addition, since certain databases are updated slowly, it is difficult to acquire more targets (Zhuang et al., 2019). To investigate the pharmacological effects of paeoniflorin in more depth, we downloaded the Microarray data of pancreatic cancer from the GEO database and screened its targets using the WGCNA method in this work. The genes in the modules screened by WGCNA were crossed with DEGs, and a total of 182 genes were obtained as target genes. KEGG and GO enrichment analysis were performed on these genes. The findings of the GO analysis suggested that epidermal growth and serine were crucial for PF intervention in pancreatic cancer. Serine promotes protein, amino acid, and glutathione production, which are critical for cell development and survival. Many tumor cells show dependence on exogenous serine and dietary serine (Yang and Vousden, 2016; Tajan et al., 2021). Pancreatic ductal adenocarcinoma patients had lower blood levels of essential and non-essential amino acids. PGAM1 knockdown increases 3-PG accumulation in serine-starved PDAC cells, resulting in increased cell proliferation and tumor formation (Itoyama et al., 2021). As for the traditional EGFR receptor, it is often referred to as ERBB-1. The MAPK pathway robustly stimulates the transcription and release of numerous ERBB ligands in pancreatic cancer (Mendelsohn and Baselga, 2003). Tumor-specific targeted delivery of 5FU using EGFR aptamers as the carrier achieved high target specificity, overcame 5 FU resistance (Mahajan et al., 2021). The KEGG analysis supports this conclusion. According to KEGG analysis, cancer MAPK signaling, PI3K-Akt signaling, and RAS signaling pathways could be regulated by PF. These pathways are intrinsically associated with the inflammatory response. Previous research has demonstrated that inflammation is present at every stage of tumor development (Greten and Grivennikov, 2019). In recent years, several endeavors have been made to discover the processes behind inflammation-induced carcinogenesis (Hausmann et al., 2014). Similarly, the GESA enrichment results were significantly correlated with GO and KEGG analyses, which were both enriched in inflammation-related aspects. Meanwhile, through algorithmic screening, we obtained 12 hub genes. The survival analysis of these genes indicated that the high expression of four of them (HBEGF, MMP1, MMP7, and MMP14) was indicative of a poor prognosis for overall survival. MMP1, MMP7, and MMP14 are all members of the family of Matrix Metalloproteinases. The MMP family drives tumor invasion and the creation of distant metastases, and is recognized to play a crucial role in a number of human malignancies, including pancreatic cancer (Huang et al., 2018). Expression of MMP1 in the early stages of a number of malignancies is correlated with a dismal prognosis. By activating MAPK pathways, RAS oncogenes may play a crucial role in the constitutive production of MMP1 in human pancreatic cancer cells (Huang et al., 2018, 1). MMP7 is involved in the injury response of mucosal epithelia and the degradation of extracellular matrix components, and it has been shown to be overexpressed in pancreatic ductal adenocarcinoma and its precursors, PanIN and intraductal papillary mucinous neoplasms, with MMP7 alterations evident even in intermediate-grade (Kartsonaki et al., 2022). IL-17 stimulates MMP7 expression in prostate cancer to destroy the E-cadherin/β-catenin complex and release -catenin, hence promoting EMT and tumor cell invasion (Zhang et al., 2017). Additionally, exosome-transferred MMP14 is a crucial facilitator of gemcitabine resistance in pancreatic cancer (Li et al., 2022).

Based on the results of CCK-8 and early studies, it was observed that PF intervenes in pancreatic cancer cells in a concentration-dependent way (in the range of 0–200 μm) in this experiment; hence, the 0–200 μm concentration was selected for further investigations. The presence of the IL-10 cytokine in the culture supernatant of PANC-1 cells was barely detectable in the first ELISA tests. This corroborates the findings of Graziella et al. (G et al., 2006). Capan-2 cells that released substantially higher IL-10 were therefore chosen. Then, we subjected PANC-1 and Capan-2 cells to 50, 100, and 200 μm dosages for 24 h and measured the levels of the cytokines IL-10 and IL-6, as well as the expression of MAPK pathway-related proteins. The results suggest that PF can have a therapeutic function by reducing the activation of inflammatory pathways and variables associated with pancreatic cancer. The activation of inflammatory signals can release a large number of cytokines, inflammatory mediators, and free radicals. Genes of the Interleukin 10 (IL-10) family play a dual, contentious function in a variety of cancers. According to a number of studies, an increase in IL-10 levels can dramatically drive tumor proliferation, metastasis, and immune evasion in a range of tumor models, including pancreatic cancer, and hence result in diverse pathologies (Zhuang et al., 2019). Our studies demonstrated that paeoniflorin dose-dependently inhibits IL-10 expression in pancreatic cancer cells. In addition, there is a complicated interaction effect between IL-10 and the MAPK pathway, whereby the activation of the MAPK pathway, which increases the production of IL-10 and TGF by cancer cells by maturing immune suppressive regulatory CD4^+^ T-cells, results in a complex interaction effect (Hou et al., 2020). The activation of an ERK/JNK/P38 MAPK inflammatory pathway by IL-10 appears to enhance the progression of chronic pancreatitis to pancreatic cancer. IL-6 can promote EMT by activating numerous molecular pathways of the JAK2/STAT3 and MAPK signaling axis, hence enhancing the metastatic potential of tumors (Abaurrea et al., 2021). In addition, our enrichment analysis revealed that the signaling pathways PI3K-AKT, and MAPK, etc. were related to pancreatic cancer. Since our hub genes are closely related to MAPK signaling pathway. Therefore, we chose MAPK signaling pathway for validation. Phosphorylation activates members of the Mitogen-activated protein kinase (MAPK) family, which includes ERK1/2, JNK, and p38. This activation causes the expression of target genes. Under normal circumstances, the expression of each member of the MAPK family maintains a dynamic balance, thereby maintaining cell proliferation and apoptosis in a balanced state. More and more studies have shown that the occurrence and development of tumors are related to the regulation of MAPK information transmission. In our study, paeoniflorin inhibited p38 but not ERK or JNK to affect pancreatic cancer. Wang et al. showed that p38gamma-MAPK promotes pancreatic cancer by activating PFKFB3 and GLUT2 through the KRAS oncogene signaling and aerobic glycolysis (Wang et al., 2020).

Nonetheless, we must recognize that PF is an unstable, water-soluble monoterpene glycoside that is strongly impacted by strong alkali and high temperatures. Therefore, when it is used in the body, whether it will have a decomposition reaction or *in volve* more complex pathways to play a role requires further research.

## 5 Conclusion

In conclusion, our research implemented a combination of bioinformatics, network pharmacology, and *in vitro* experiments to analyze the potential PF treatment pathways for pancreatic cancer. PF inhibits the proliferation of pancreatic cancer cells by interfering with the MAKP signaling pathway, IL-10, IL-6, and additional inflammatory factors. We provide some references for the development of PF as a follow-up treatment for pancreatic cancer.

## Data Availability

Publicly available datasets were analyzed in this study. This data can be found here: GSE97124, https://www.ncbi.nlm.nih.gov/geo/; The Cancer Genome Atlas, https://portal.gdc.cancer.gov/; Genotype-Tissue Expression, https://commonfund.nih.gov/GTEx.
