# Supplementary material for: Deciphering the action mechanism of paeoniflorin in suppressing pancreatic cancer: A network pharmacology study and experimental validation
Source: Front Pharmacol. 2022 Oct 20;13:1032282. doi: 10.3389/fphar.2022.1032282 (PMC9630940; doi:10.3389/fphar.2022.1032282)
Supplement: Supplementary file 1 [file DataSheet1.docx]

**SUPPLEMENTARY MATERIAL**
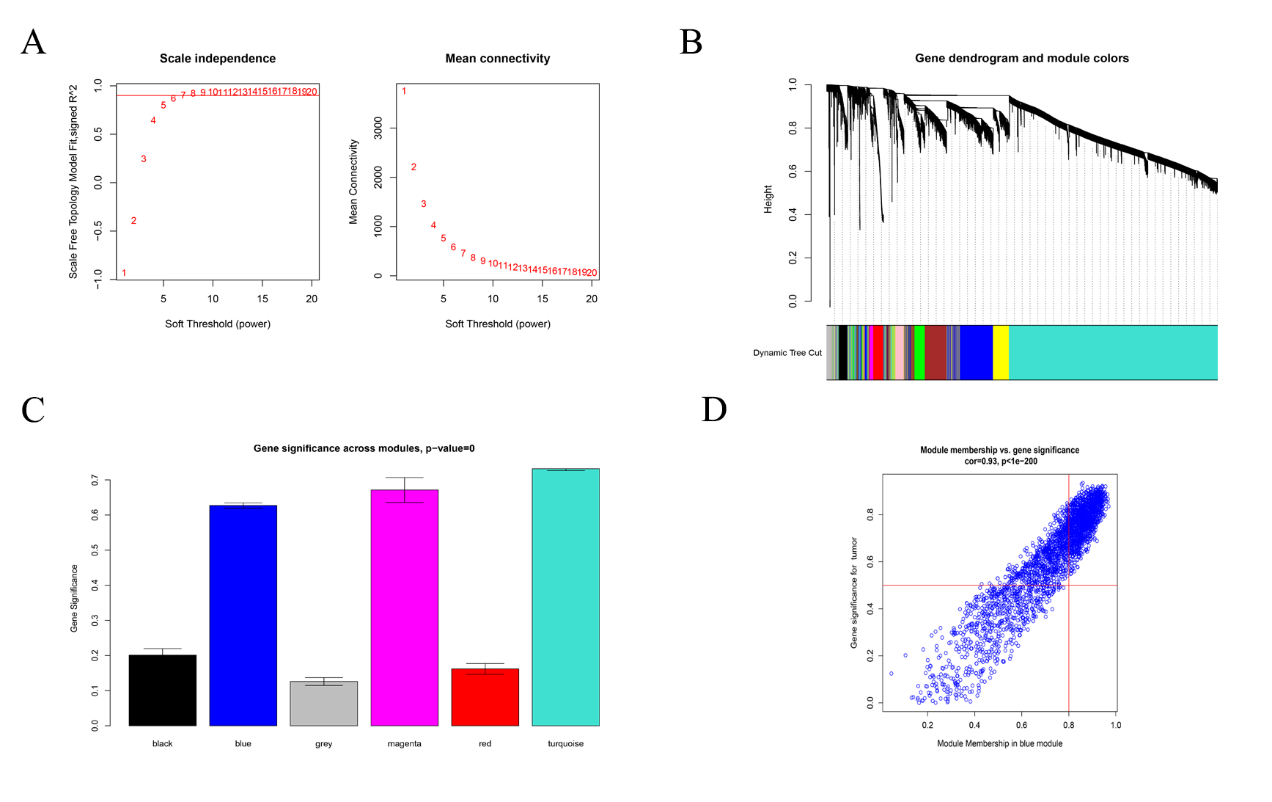


Fig.S1 WGCNA analysis

(A) Both scale independence (left) and Mean connectedness (right) can yield superior outcomes when the soft threshold is equal to 9. (B) A hierarchical clustering tree comprising 22 modules of co-expressed genes is displayed. Each of the 6 modules is shown by a branch with a different color. (C) Gene significance across modules. (D) Relationships between modules and corresponding P-values.

Table S1 Top 10 enriched entries by P-value in GO enrichment analysis

| ONTOLOGY | ID | Description | GeneRatio | BgRatio | pvalue |
| --- | --- | --- | --- | --- | --- |
| BP | GO:0042058 | regulation of epidermal growth factor receptor signaling pathway | 8/170 | 89/18862 | 1.40E-06 |
| BP | GO:1901184 | regulation of ERBB signaling pathway | 8/170 | 96/18862 | 2.50E-06 |
| BP | GO:0045741 | positive regulation of epidermal growth factor-activated receptor activity | 4/170 | 13/18862 | 4.27E-06 |
| BP | GO:0032963 | collagen metabolic process | 8/170 | 109/18862 | 6.47E-06 |
| BP | GO:0007176 | regulation of epidermal growth factor-activated receptor activity | 5/170 | 30/18862 | 6.66E-06 |
| BP | GO:1901185 | negative regulation of ERBB signaling pathway | 6/170 | 53/18862 | 7.93E-06 |
| BP | GO:0060323 | head morphogenesis | 5/170 | 33/18862 | 1.08E-05 |
| BP | GO:0007173 | epidermal growth factor receptor signaling pathway | 8/170 | 123/18862 | 1.58E-05 |
| BP | GO:0050679 | positive regulation of epithelial cell proliferation | 10/170 | 203/18862 | 1.59E-05 |
| BP | GO:0042058 | regulation of epidermal growth factor receptor signaling pathway | 8/170 | 89/18862 | 1.40E-06 |
| CC | GO:0005788 | endoplasmic reticulum lumen | 13/174 | 306/19520 | 3.81E-06 |
| CC | GO:0005765 | lysosomal membrane | 13/174 | 378/19520 | 3.59E-05 |
| CC | GO:0098852 | lytic vacuole membrane | 13/174 | 378/19520 | 3.59E-05 |
| CC | GO:0030665 | clathrin-coated vesicle membrane | 7/174 | 117/19520 | 8.63E-05 |
| CC | GO:0005925 | focal adhesion | 13/174 | 416/19520 | 9.50E-05 |
| CC | GO:0030055 | cell-substrate junction | 13/174 | 423/19520 | 0.000112207 |
| CC | GO:0005774 | vacuolar membrane | 13/174 | 431/19520 | 0.000135086 |
| CC | GO:0032580 | Golgi cisterna membrane | 6/174 | 94/19520 | 0.000195899 |
| CC | GO:0030136 | clathrin-coated vesicle | 8/174 | 192/19520 | 0.000333894 |
| CC | GO:0030667 | secretory granule membrane | 10/174 | 305/19520 | 0.000423391 |
| MF | GO:0008236 | serine-type peptidase activity | 10/175 | 186/18337 | 1.22E-05 |
| MF | GO:0017171 | serine hydrolase activity | 10/175 | 188/18337 | 1.34E-05 |
| MF | GO:0004252 | serine-type endopeptidase activity | 9/175 | 168/18337 | 3.45E-05 |
| MF | GO:0005178 | integrin binding | 8/175 | 142/18337 | 6.64E-05 |
| MF | GO:0005520 | insulin-like growth factor binding | 4/175 | 29/18337 | 0.000157986 |
| MF | GO:0005154 | epidermal growth factor receptor binding | 4/175 | 33/18337 | 0.000264214 |
| MF | GO:0004175 | endopeptidase activity | 13/175 | 438/18337 | 0.000305393 |
| MF | GO:0004222 | metalloendopeptidase activity | 6/175 | 107/18337 | 0.000565254 |
